# Do you want to live forever? Lessons learned from the biology of aging

**DOI:** 10.1371/journal.pbio.3003835

**Published:** 2026-05-29

**Authors:** William B. Mair, Ines Alvarez-Garcia

**Affiliations:** 1 Department of Molecular Metabolism, Harvard TH Chan School of Public Health, Boston, Massachusetts, United States of America; 2 Public Library of Science, San Francisco, California, United States of America, and Cambridge, United Kingdom

## Abstract

Aging affects us all, but we still do not know how the process evolves or if we can modulate its pace. This Editorial presents a Collection of articles that explores different aspects of aging, discussing what challenges still need to be overcome.

## Teaser

Aging affects us all, but we still do not know how the process evolves or if we can modulate its pace. This issue of *PLOS Biology* presents a Collection of articles that explores different aspects of aging, discussing what challenges still need to be overcome.

Throughout history, humans have been fascinated by their own mortality, seeking ways to delay or even reverse the ravages of time. However, there has been an unprecedented explosion of public interest in ‘longevity’ in the past 5 years, driven both by evidence-based advances in geroscience [1] and by hyperbolic claims by so-called ‘wellness influencers’ on social media. As biomedical companies seek to translate pre-clinical discoveries in precision geroscience into therapeutics, the field stands at an inflection point. That biological age can be both measured and modulated to target age-related disease and compress late-life morbidity is now broadly accepted. Yet, to prove its worth, aging research needs a win. The exciting news is that we have never been closer to that win, with clinical trials of interventions born in geroscience labs already underway [2]. Scaling geroscience discovery to geromedical interventions will not be easy, requiring robust biomarkers and outcomes that withstand the heterogeneity of human populations and their exposures. In this issue of *PLOS Biology*, we present a Collection of articles that highlight areas of opportunity and caution in the aging field that can collectively help to guide us as we take on this 21st-century challenge.

The global average life expectancy is now ~73.8 years at birth, a striking increase over the 51–53-year life expectancy of the 1960s [3]. However, there is a common misconception that increased longevity translates into slower aging. In this issue, Modig and Ebeling [4] discuss the likelihood that the increase is mainly due to the success of public health and modern medicine in helping humans avoid or survive most diseases, rather than a true modification of the biological aging process. This is important to understand in a changing world that has a greater number of aged individuals with chronic conditions who are in need of long-term treatments; simply extending life, rather than improving it, is not good enough. One of the hallmarks of aging is cognitive decline, which can affect memory and learning. Mhatre and Moore [5] explore the possibility of limiting cognitive aging by increasing the neural stem cell population in the human hippocampus. They discuss the fact that adult neurogenesis takes place in humans and that so-called ‘SuperAgers’ have a higher number of newborn neurons compared with aged adults. Might it then be possible to have ‘healthy’ aging? Alam, Partridge, and Alic [6] believe that interventions that slow aging are possible and can have long-term effects if applied in early adulthood, constituting a ‘physiological memory’. They argue that the study of the biology of aging using model organisms has provided a significant shift in our understanding of the mechanisms that allow individuals to improve their health in older age. It is becoming very clear that nutrient signaling pathways are fundamental factors from the point of conception or even before, as signaling states can be imprinted, and that defining physiological memory may be key to delivering individual anti-aging therapies.

Can we push our known limits and dream about becoming younger? Chiavellini and Sebastiano [7] ask this question, reasoning that because mammalian ovaries are sites of natural restoration to a youthful state within an aging organism, ovarian biology is an ideal model to study rejuvenation. Their Essay discusses how we can learn from the various cellular processes, including epigenetic reprogramming, mitochondrial quality control, and proteostasis, that cooperate in the oocyte (along with tissue homeostasis mechanisms within the ovarian niche) to sustain youth. An exciting possibility they consider is that the ovary could serve as a rejuvenation hub and influence other tissues and organs, beyond fertility. The Perspective by Jennifer Garrison [8] provides a refreshing view on how the ovaries are more than mere egg factories, connecting with most tissues in the body through hormones and other factors that circulate in the blood to maintain health. This raises the possibility that ovarian signaling is disrupted across tissues decades before menopause starts. Thus, instead of focusing ovarian aging research around menopause, perhaps we should look much earlier.

Old age is also strongly characterized by failures of the immune system that accumulate over time, driving the aging process and promoting age-related diseases. Two pieces in the Collection analyze the influence of the aging immune system. Baker, Benayoun, and colleagues [9] focus on the influence of biological sex on ‘immunosenescence’—the decline of the immune system with age—and ‘inflammaging’—old age-associated chronic inflammation. Indeed, there are striking differences in both the timing and slope of the trajectories of immune capacity throughout life in men and women that must be considered for personalized medical interventions. The Unsolved Mystery by Liu, Costa, and Valenzano [10] raises several questions regarding the collapse of host-associated microbiomes during aging, including what starts this process and why stability fails. The authors propose that lifelong immune surveillance is essential for immune–microbiome interactions, constraining microbial proliferation and inhibiting community compositional drift. Could it be that age-associated microbiome dysbiosis is not a passive collapse, but the failure of an effective surveillance system that maintains a healthy community? If so, it is possible that restoring the composition of these ecosystems could slow the aging process.

While the public’s excitement about longevity makes the mission to slow or even reverse aging one that many people share, trust in science and in companies that convert foundational discoveries into for-profit medical interventions is strained. Yet, this in itself is also an opportunity. Aging affects every one of us, and all solutions, be they peptides or policies, will need to be grounded in evidence and accessible to all. The fundamental principles of geroscience lie in disease prevention and in maintaining good health into old age. These are core goals shared by all of us, no matter our political beliefs or how much we trust research. Achieving them may well require geroprotecting drugs, but might equally leverage nutrition, exercise physiology, environmental exposure, probiotics, chronobiology, or social and behavioral science. What will be important is not the source of the geroscience win, but its efficacy, safety, and how soon it is delivered. The goal of this Collection is to help catalyze that process.
